# Induction and Repression of Hydrolase Genes in *Aspergillus oryzae*

**DOI:** 10.3389/fmicb.2021.677603

**Published:** 2021-05-24

**Authors:** Mizuki Tanaka, Katsuya Gomi

**Affiliations:** ^1^Biomolecular Engineering Laboratory, School of Food and Nutritional Science, University of Shizuoka, Shizuoka, Japan; ^2^Laboratory of Fermentation Microbiology, Graduate School of Agricultural Science, Tohoku University, Sendai, Japan

**Keywords:** *Aspergillus oryzae*, hydrolase, carbon catabolite repression, transcription factor, ubiquitination, endocytosis

## Abstract

The filamentous fungus *Aspergillus oryzae*, also known as yellow *koji* mold, produces high levels of hydrolases such as amylolytic and proteolytic enzymes. This property of producing large amounts of hydrolases is one of the reasons why *A. oryzae* has been used in the production of traditional Japanese fermented foods and beverages. A wide variety of hydrolases produced by *A. oryzae* have been used in the food industry. The expression of hydrolase genes is induced by the presence of certain substrates, and various transcription factors that regulate such expression have been identified. In contrast, in the presence of glucose, the expression of the glycosyl hydrolase gene is generally repressed by carbon catabolite repression (CCR), which is mediated by the transcription factor CreA and ubiquitination/deubiquitination factors. In this review, we present the current knowledge on the regulation of hydrolase gene expression, including CCR, in *A. oryzae*.

## Introduction

The *koji* mold *Aspergillus oryzae* is a filamentous fungus that has been used for over a thousand years to manufacture Japanese fermented foods and beverages, such as shoyu (soy sauce), miso (soybean paste), and sake (rice wine) (Machida et al., 2008). The most industrially important characteristic of *A. oryzae* is the ability to produce large amounts of hydrolytic enzymes such as amylolytic and proteolytic enzymes. This is one of the reasons why *A. oryzae* has been used in the production of traditional Japanese fermented foods and beverages. Genome sequencing of *A. oryzae* has revealed that this fungus has more hydrolytic enzyme genes than other related filamentous fungi such as *Aspergillus nidulans* and *Aspergillus fumigatus* (Machida et al., 2005; Kobayashi et al., 2007). A wide variety of hydrolases produced by *A. oryzae* have been used in the food processing and pharmaceutical industries, in addition to the fermentation industry (Gomi, 2014). Moreover, promoters of hydrolytic enzyme genes, especially those of amylolytic genes, are widely used for high expression of homologous and heterologous genes in *A. oryzae* to produce useful proteins and secondary metabolites (Tanaka and Gomi, 2014; Oikawa, 2020; Jin et al., 2021). Therefore, understanding the regulation of hydrolytic enzyme gene expression in *A. oryzae* is both scientifically and industrially important. The expression of most hydrolase genes is induced by the presence of certain substrates. For instance, it has long been known that amylolytic gene expression is induced by starch and malto-oligosaccharides (Tonomura et al., 1961; Yabuki et al., 1977). Such substrate-specific gene expression is often regulated by fungal-specific Zn(II)_2_Cys_6_-type transcription factors. Several transcription factors that control the expression of hydrolytic enzyme genes in *A. oryzae* have been identified (Table 1), and the regulatory mechanisms of their activation have recently been elucidated. On the other hand, in the presence of glucose, the expression of hydrolytic enzyme genes is strongly repressed even when inducing substrates are present. This phenomenon is known as carbon catabolite repression (CCR). CCR-regulating factors have been identified, and their function has been analyzed in the model filamentous fungus *A. nidulans*; however, details on the control mechanism of CCR remain unclear. Recent studies have revealed a part of the molecular mechanism of CCR regulation in *A. oryzae*.

**Table 1:** Transcription factors involved in induction of hydrolytic gene expression in *Aspergillus oryzae*.

Transcription factor	DNA-binding motif	Major regulated genes
AmyR	Zn(II)_2_Cys_6_-type	Amylolytic genes (*amyA*, *amyB*, *amyC*, *glaA*, *glaB*, and *agdA*)
MalR	Zn(II)_2_Cys_6_-type	Maltose transporter gene (*malP*), maltase gene (*malT*)
FlbC	C_2_H_2_-type	Solid-state culture-specific expression genes (*glaB* and *pepA*), neutral protease gene (*nptB*)
XlnR	Zn(II)_2_Cys_6_-type	Xylolytic genes (*xynF1*, *xynG1*, *xynG2*, and *xylA*), cellulolytic genes (*celC*, *celD*, *cbhD*, and *bgl5*), pentose catabolic enzyme genes (*xyrA*, *ladA*, and *xdhA*), and putative xylose transporter genes
AraR	Zn(II)_2_Cys_6_-type	Pentose catabolic enzyme genes (*larA*, *xyrA*, *ladA*, and *xdhA*)
ManR	Zn(II)_2_Cys_6_-type	Mannanolytic genes (*manD*, *manG*, *mndB*, and *mndD*), cellulolytic genes (*celC*, *celD*, *cbhD*, and *bgl5*)
PrtR	Zn(II)_2_Cys_6_-type	Proteolytic genes (*alpA*, *pepA*, *nptA*, *nptB*, and *tppA*), di/tripeptide transporter genes (*potA*, *potB*)
FarA	Zn(II)_2_Cys_6_-type	Cutinase-like lipase gene (*cutL1*), hydrophobin genes (*rolA* and *hsbA*)

In this review, we introduce transcription factors that induce the expression of hydrolytic enzyme genes in *A. oryzae*. In addition, we describe the molecular mechanism of CCR, which was revealed by recent studies on *A. oryzae* and other filamentous fungi.

## Transcription Factors That Induce Gene Expression of Hydrolytic Enzymes in *A. Oryzae*

### AmyR and MalR

The regulation of amylolytic gene expression in *A. oryzae* has been studied for many years, because amylolytic enzymes are the major hydrolytic enzymes produced by *A. oryzae*, and are essential for sake production (Gomi, 2019). The expression of amylolytic genes is directly regulated by the transcription factor AmyR (Petersen et al., 1999; Gomi et al., 2000). This Zn(II)_2_Cys_6_-type transcription factor binds to the CGGN_8_(C/A)GG sequence in the promoter of amylolytic genes, such as the α-amylase (*amyA/B/C*), glucoamylase (*glaA* and *glaB*), and α-glucosidase (*agdA*) genes (Petersen et al., 1999; Ito et al., 2004). Expression of these genes is induced by maltose and isomaltose, and also by glucose when CCR is released (Suzuki et al., 2015). The *amyR* gene is constitutively expressed regardless of the presence of inducing or non-inducing sugars, and under conditions where amylolytic genes are not expressed; AmyR is localized in the cytoplasm (Suzuki et al., 2015). AmyR is rapidly transferred into the nucleus when isomaltose is added to the medium (Suzuki et al., 2015). Glucose and maltose also induce nuclear transfer of AmyR, but these sugars require higher concentrations and longer time periods than isomaltose to induce AmyR nuclear transfer and amylolytic gene expression (Suzuki et al., 2015). This dynamic of AmyR nuclear transfer is similar to that in the model filamentous fungus *A. nidulans* (Murakoshi et al., 2012). The nuclear transfer of AmyR depends on nuclear localization signals located within its DNA-binding domain (Suzuki et al., 2015). In contrast, C-terminal truncated AmyR is constitutively localized in the nucleus in both *A. nidulans* and *A. oryzae* (Makita et al., 2009; Suzuki et al., 2015). This suggests that the C-terminal region of AmyR is required to keep AmyR in the cytoplasm. C-terminal truncated *A. nidulans* AmyR retains its transcriptional activation function (Makita et al., 2009), but C-terminal truncation of *A. oryzae* AmyR leads to the loss of such function (Suzuki et al., 2015). This suggests that the function of the C-terminal region of AmyR differs among *Aspergillus* species. Therefore, the function of the C-terminal region of AmyR in other *Aspergillus* species that produce large amounts of amylolytic enzymes, such as *Aspergillus niger* and *Aspergillus luchuensis*, should be investigated.

In *A. nidulans*, maltose utilization is dependent on AmyR (Tani et al., 2001). However, a disruption mutant of *A. oryzae amyR* was able to assimilate maltose because maltose utilization in *A. oryzae* is regulated by another Zn(II)_2_Cys_6_-type transcription factor, MalR (Hasegawa et al., 2010). MalR is a homolog of the yeast *MAL* activator, a transcriptional activator of genes encoding maltose transporter and maltase (Needleman, 1991). In contrast to AmyR, MalR is constitutively localized in the nucleus (Suzuki et al., 2015). Similar to the yeast *MAL* activator gene, *malR* comprises a gene cluster with the maltase gene (*malT*) and maltose transporter gene (*malP*), and the expression of both these genes is regulated by MalR (Hasegawa et al., 2010). The expression of *malP* and *malT* is not induced by the addition of isomaltose, but is preceded by amylolytic gene expression upon the addition of maltose (Suzuki et al., 2015). Therefore, MalR is activated prior to AmyR to convert maltose into a physiologically active inducing substrate for AmyR, i.e., isomaltose. This conversion of maltose to isomaltose is probably caused by the glycosyltransferase activity of MalT. In budding yeast, activation of the *MAL* activator is regulated by the dissociation of its chaperone proteins Hsp70 and Hsp90 (Ran et al., 2008). Although *A. oryzae* MalR interacts with orthologs of Hsp70 and Hsp90 (Konno et al., 2018), details on the activation mechanism and binding sequence are not known.

Importantly, the α-amylase (*amyB*) gene promoter is commonly used for high-level expression of heterologous genes in *A. oryzae* (Jin et al., 2021). Improved promoters *PglaA142* (Minetoki et al., 2003) and *PenoA142* (Tsuboi et al., 2005), have been constructed by tandem insertion of AmyR-binding sequences (Region III) within the glucoamylase and enolase gene promoters. These improved promoters are now utilized in homologous and heterologous protein production.

### FlbC

*Aspergillus oryzae* has two glucoamylase genes, *glaA* and *glaB*, the expression patterns of which are quite different (Hata et al., 1991, 1998). Similar to other amylolytic enzymes, GlaA is produced in both submerged and solid-state cultures (Oda et al., 2006). On the other hand, GlaB is secreted exclusively in solid-state culture and is not produced in submerged culture. Although the expression of both *glaA* and *glaB* is regulated by AmyR (Watanabe et al., 2011), a transcription factor that binds to the promoter region of *glaB* and regulates the expression of specific genes in solid-state culture was expected to be present (Ishida et al., 2000; Hisada et al., 2013). A screening of the *A. oryzae* disruption mutant library for transcriptional regulators indicated that FlbC is a transcription factor that regulates the expression of *glaB* (Tanaka et al., 2016). In addition to that of *glaB*, the expression level of *pepA*, an aspartic protease gene that is predominantly expressed in solid-state culture, was also significantly reduced by *flbC* disruption (Tanaka et al., 2016). Therefore, FlbC is presumed to be a transcription factor that regulates the specific expression of hydrolytic enzyme genes in solid-state culture. The expression of the neutral protease gene (*nptB*) is also regulated by FlbC (Tanaka et al., 2016). FlbC is a C_2_H_2_-type transcription factor that was originally identified as one of the regulators of conidiospore development (Wieser et al., 1994; Kwon et al., 2010; Ogawa et al., 2010). Disruption of other transcription factors that control conidiospore development has no effect on the production of GlaB, suggesting that FlbC regulates *glaB* expression independently of the regulatory mechanism of conidiospore development (Tanaka et al., 2016). Moreover, FlbC probably binds directly to the promoter region of *glaB* and regulates its expression (Gomi, 2019). Although the FlbC-binding sequence has not been empirically identified, the sequence containing GATC would be a candidate based on recent studies of the FlbC orthologs of *Neurospora crassa* (Boni et al., 2018) and *Magnaporthe oryzae* (Matheis et al., 2017). Furthermore, the regulatory mechanism of specific gene expression in solid-state culture should be elucidated. Expression from the *glaB* promoter is induced by low water activity, high temperature, and physical barriers to hyphal extension (Ishida et al., 1998). Since these inducing factors cause environmental stress in *A. oryzae* cells, the involvement of stress response pathways, including that of mitogen-activated protein kinase (MAPK) signaling pathways, in FlbC activation should be considered.

### XlnR and AraR

XlnR is a Zn(II)_2_Cys_6_-type transcription factor that regulates the expression of xylanolytic and cellulolytic genes (Marui et al., 2002). This transcription factor is also involved in the regulation of putative xylose transporter genes and pentose metabolic enzyme genes (Noguchi et al., 2009). In *Aspergillus* species, the expression of pentose catabolic pathway genes is also regulated by AraR, a paralog of XlnR (Battaglia et al., 2011). Electrophoretic mobility shift assays revealed that XlnR and AraR bind competitively to the CGGNTAAW sequence in the promoter region of pentose catabolic genes such as the xylose dehydrogenase-encoding gene (*xdhA*) (Ishikawa et al., 2018). Notably, XlnR binds to the CGGNTAAW sequence solely found in the promoter region of pentose catabolic genes as a monomer, whereas it binds to the TTAGSCTAA and TAGSCTA sequences in the promoter region of the xylanase genes (*xynF1* and *xynG2*) as a dimer (Ishikawa et al., 2018).

XlnR is constitutively located in the nucleus, similar to MalR. When xylose is added, XlnR is rapidly phosphorylated. In contrast, XlnR is rapidly dephosphorylated when xylose is removed from the culture medium (Noguchi et al., 2011). This reversible phosphorylation probably regulates the activation of XlnR. Identification of the phosphorylation site of XlnR is important for elucidating the activation mechanism of this transcription factor.

### ManR

ManR is a Zn(II)_2_Cys_6_-type transcription factor that regulates the expression of mannanolytic enzyme genes. This transcription factor was identified by screening for mutants exhibiting reduced β-mannanase activity from a gene disruptant library of transcriptional regulators (Ogawa et al., 2012). ManR also regulates cellulolytic enzyme genes, such as the cellobiohydrolase, endoglucanase, and β-glucosidase genes (Ogawa et al., 2013). Most of these genes are also regulated by XlnR. Therefore, ManR and XlnR probably regulate the expression of these genes in a coordinated manner (Tani et al., 2014). ManR is an ortholog of *N. crassa* CLR-2 and *A. nidulans* ClrB, both of which regulate the expression of cellulase genes. However, the physiological roles of these transcription factors are slightly different (Kunitake and Kobayashi, 2017). ManR binds to the promoter regions of the β-mannanase gene (*manG*), which contains the CAGAAT sequence that is conserved in the promoter regions of mannanolytic enzyme genes (Ogawa et al., 2012). However, this conserved sequence is quite different from consensus sequences for the binding of CLR-2 and ClrB (Kunitake and Kobayashi, 2017).

### PrtR

PrtR is a Zn(II)_2_Cys_6_-type transcription factor; the deletion of this transcription factor results in a significant decrease in extracellular protease activity (Mizutani et al., 2008), indicating that it is essential for extracellular proteolytic gene expression. Interestingly, the *prtR* gene is located adjacent to the amylolytic gene cluster consisting of *amyR*, *agdA*, and *amyA* (Gomi, 2019). Orthologs of PrtR in other *Aspergillus* species are named PrtT (Punt et al., 2008). Duplication or triplication of the chromosomal region containing the *prtR* gene by forced translocation of the 1.4 Mb chromosome 2 resulted in a significant increase in alkaline protease and acid carboxypeptidase activity in a solid-state culture of *A. oryzae* (Takahashi et al., 2018). Over 20 proteolytic genes were upregulated in the duplicated strain. Furthermore, the expression level of an alkaline protease gene (*alpA*) increased more than 5-fold when *prtR* was highly expressed from the promoter of the α-amylase gene (Takahashi et al., 2018). In addition, the expression levels of two of the three di/tripeptide transporter genes (*potA* and *potB*) also increased upon *prtR* overexpression and decreased upon *prtR* disruption (Tanaka et al., 2021). These results suggest that PrtR plays a central role in the regulation of gene expression for the acquisition of nutrients in the presence of protein. In fact, a disruption mutant of *prtR* showed poor growth in solid-state culture using wheat bran as a substrate. In *A. niger* and *A. fumigatus*, PrtT regulates the expression of multiple protease genes and tri/tetrapeptide transporter genes (Sharon et al., 2009; Hartmann et al., 2011; Huang et al., 2020); PrtT orthologs are absent in *A. nidulans* (Punt et al., 2008). Although the expression of proteolytic genes is induced by proteins or peptides, the direct substrate that induces the activation of PrtR/PrtT is not clear for any *Aspergillus* species; hence, further studies are required to ascertain this. Disruption of multiple protease genes has resulted in highly effective heterologous protein production in *A. oryzae* (Jin et al., 2021). Considering that many extracellular protease genes are thought to be regulated by PrtR, the effect of *prtR* disruption on heterologous protein production is strongly expected.

### FarA

*Aspergillus oryzae* can degrade polyester poly(butylene succinate-*co*-adipate) (PBSA), a biodegradable plastic. The cutinase-like lipase CutL1 is the major enzyme that degrades PBSA in *A. oryzae*, and two hydrophobic surface binding proteins (RolA and HsbA) assist in the binding of CutL1 to PBSA (Maeda et al., 2005; Takahashi et al., 2005; Ohtaki et al., 2006). FarA is a Zn(II)_2_Cys_6_-type transcription factor that regulates the expression of fatty acid metabolism genes in *A. nidulans*; its ortholog, CTF1α of *Fusarium solani*, regulates cutinase gene expression (Li and Kolattukudy, 1997; Hynes et al., 2006). The expression of the *cutL1* gene is regulated by FarA, and a disruption mutant of *farA* abolished PBSA degradation activity in *A. oryzae* (Garrido et al., 2012). In addition to that of the *cutL1* gene, the expression of *rolA* and *hsbA* is also repressed by the disruption of *farA* (Garrido et al., 2012). Although details on the mechanism of FarA regulation of the cutinase gene are unknown for all *Aspergillus* species, a recent study on *A. nidulans* showed that FarA-dependent expression of the cutinase genes is affected by CCR (Bermúdez-García et al., 2019).

## Carbon Catabolite Repression of Hydrolytic Enzyme Genes

### Regulating Factors of CCR

The study of CCR in filamentous fungi began with the identification of regulatory factors in *A. nidulans*. Four factors involved in the regulation of CCR in filamentous fungi were identified in the 1970s by genetic analysis of *A. nidulans* (Arst and Cove, 1973; Hynes and Kelly, 1977; Kelly and Hynes, 1977). These four factors were denoted as CreA, CreB, CreC, and CreD. Firstly, the *creA* gene was identified in an *A. nidulans* CCR-deficient mutant. This gene encodes a C_2_H_2_-type transcription factor that directly regulates CCR (Dowzer and Kelly, 1991). Similar to *A. nidulans* CreA, *A. oryzae* CreA binds to the SYGGRG sequence in the promoter region of the α-amylase gene (Kato et al., 1996). The amino acid sequence of the CreA DNA-binding domain is highly homologous to that of Mig1, a CCR-regulating transcription factor in the budding yeast *Saccharomyces cerevisiae*. However, the homology between CreA and Mig1 in the regions other than the DNA-binding domain is not high. In addition, recent studies have revealed that the functional control mechanisms of Mig1 and CreA are quite different (see Nuclear export-dependent degradation of CreA).

Similar to *creA* (described above), *creB* and *creC* were identified in CCR-deficient mutants as well. CreB is a deubiquitinating enzyme homolog with a particularly high homology to Ubp9 of the fission yeast *Schizosaccharomyces pombe* (Lockington and Kelly, 2001). Many deubiquitinating enzymes interact with WD40-repeat proteins that regulate the activity and function of such enzymes (Villamil et al., 2013). The *creC* gene encodes a WD40-repeat protein that shows high homology to fission yeast Bun62 (Lockington and Kelly, 2002) but has no counterpart in budding yeast. In *A. nidulans*, CreB and CreC form complexes *in vivo* (Lockington and Kelly, 2002). In fission yeast, Ubp9 interacts with Bun62 and another WD40-repeat protein, Bun107, and is involved in endocytosis, actin dynamics, and cell polarity (Kouranti et al., 2010). However, the function of the CreB and CreC complex in filamentous fungi has not been clarified.

The gene responsible for suppressing the phenotype of *creB* and *creC* mutants was identified and named *creD* (Kelly and Hynes, 1977). CreD contains two arrestin domains and three or four PxY motifs that are involved in protein-protein interactions (Boase and Kelly, 2004). There is a high homology between CreD and yeast Rod1/Art4, an arrestin-related trafficking adaptor (ART) protein that acts as an adaptor for ubiquitin ligase and its target protein. In budding yeast, ART proteins recruit the HECT E3 ubiquitin ligase Rsp5 to cell membrane proteins (Lin et al., 2008; Nikko and Pelham, 2009). *Aspergillus oryzae* CreD also physically interacts with HulA, an ortholog of yeast Rsp5 (Tanaka et al., 2017). CreD and HulA are involved in the degradation of the maltose transporter MalP in *A. oryzae* (see Glucose-induced endocytosis of maltose transporter).

Based on the putative function of CreB and CreD, a model was proposed wherein CCR is regulated by the stabilization and degradation of CreA protein mediated by these factors (Lockington and Kelly, 2002; Boase and Kelly, 2004). However, this hypothesis was not supported by several recent studies (see Nuclear export-dependent degradation of CreA).

### Improved Production of Hydrolytic Enzymes by Disruption and Mutation of CCR Regulators

The release of CCR is highly effective in improving hydrolytic enzyme production in filamentous fungi. For instance, disruption and mutation of the *creA* ortholog in *Trichoderma reesei* significantly increase the production of cellulase (Nakari-Setälä et al., 2009). α-Amylase production in *A. oryzae* also increases upon the disruption of *creA* (Ichinose et al., 2014). Disruption of *creB* also increases the production of α-amylase in *A. oryzae* (Hunter et al., 2013; Ichinose et al., 2014). Moreover, double disruption of *creA* and *creB* further increases amylase production, which is more than 10 times higher than that in the wild-type strain (Ichinose et al., 2014). The transcript level of the α-amylase gene markedly increased in the *creA* disruption strain, whereas it only slightly increased upon *creB* disruption (Ichinose et al., 2014). This suggests that the destruction of *creA* and *creB* has different effects on α-amylase production. In addition to those of α-amylase, the production levels of xylanase and β-glucosidase significantly increased upon the double disruption of *creA* and *creB* (Ichinose et al., 2018). In contrast, *creA* and *creB* disruption had no effect on cellulase (endo-β-glucanase) production. In *A. nidulans*, *creA* disruption did not result in de-repression of cellulose production; it was de-repressed by the disruption of protein kinase A gene (*pkaA*) (Kunitake et al., 2019), suggesting that CreA is not relevant to CCR regulation of cellulase production in *A. oryzae*.

In *A. oryzae* CreD, two serine residues at positions 402 and 515 were identified as phosphorylation sites (Tanaka et al., 2017). Mutation of these phosphorylation sites to glutamic acid for the phosphorylation mimic repressed the amylolytic enzyme production of the *creB* disruption mutant in the presence of glucose (Tanaka et al., 2017). In contrast, dephosphorylation mutations of CreD promoted CCR release by *creB* disruption and increased the production levels of α-amylase (Tanaka et al., 2017). This finding provides a novel approach, the combination of the dephosphorylation mutation of CreD and *creB* disruption, to improve the production of secretory glycoside hydrolases in filamentous fungi. In addition, these results suggest that CreB targets unknown factor(s) that are recognized by dephosphorylated CreD for ubiquitination. However, CreA is unlikely to be a target factor for CreB and CreD (see Nuclear export-dependent degradation of CreA).

### Nuclear Export-Dependent Degradation of CreA

In budding yeast, Mig1 shuttles between the nucleus and cytoplasm in response to the glucose concentration. When green fluorescent protein (GFP) is fused to CreA in *A. oryzae*, almost all GFP fluorescence is observed in the nucleus in the presence of CCR-inducing sugars such as glucose and mannose. However, when sugars such as maltose and xylose, which induce the expression of glycosyl hydrolase genes, are used as carbon sources, CreA is exported to the cytoplasm. This nuclear export depends on a leucine-rich nuclear export signal (NES) near the C-terminus of CreA. CreA with a 3 × FLAG tag fused to its N-terminus is rapidly degraded when maltose or xylose is used as a carbon source. However, mutations in the NES significantly inhibit the degradation of CreA (Tanaka et al., 2018). These results indicate that CreA is rapidly degraded in the cytoplasm after export from the nucleus under conditions that induce the production of secretory hydrolytic enzymes. The deletion of a 20 amino acid region near the C-terminus significantly stabilizes CreA (Tanaka et al., 2018). This 20 amino acid region is highly conserved in the CreA orthologs of other filamentous fungi.

In *A. oryzae*, disruption of *creB* and *creC* significantly reduces the amount of CreA in the presence of glucose (Tanaka et al., 2018). Similarly, CreA-GFP protein levels also reduced in an *A. nidulans creC* mutant strain (Ries et al., 2016). In addition, *creA* transcript levels reduced in the presence of glucose in an *A. nidulans creB* mutant strain (Strauss et al., 1999). This reduced CreA protein level may have contributed to the release of CCR by the disruption of *creB* or *creC*. However, as mentioned above, the increase in transcript levels of the α-amylase gene by disruption of *creB* was slight, and double disruption of *creA* and *creB* resulted in a significant increase in α-amylase production (Ichinose et al., 2014), suggesting that the main reason for CCR release due to *creB* disruption is independent of the decrease in CreA abundance. In addition, CreA stability was not significantly affected by the disruption of *creD* (Tanaka et al., 2018). Therefore, there are likely to be other factor(s) involved in the regulation of CreA-independent CCR, the stability of which is controlled by CreB and CreD. Identification of such CreB and CreD target factors would lead to an understanding of CCR regulation in filamentous fungi.

In *A. nidulans*, the amount of CreA-GFP protein under repressing conditions significantly increased upon the deletion of *fbx23*, an F-box protein that constitutes the Skp-Cullin-F-box (SCF) ubiquitin ligase complex (de Assis et al., 2018). Therefore, CreA is possibly degraded in an SCF ubiquitin ligase complex-dependent manner in filamentous fungi. However, there is no direct experimental evidence for CreA ubiquitination, although the polyubiquitin precursor Ubi4 was identified as an interacting partner for both CreA and Fbx23 (de Assis et al., 2018). Further investigation of CreA degradation is required for a better understanding of the CCR regulation mechanism in filamentous fungi.

In budding yeast, Mig1 is phosphorylated by the cyclic AMP (cAMP) kinase Snf1 under low-glucose conditions, and exported from the nucleus to the cytoplasm (DeVit and Johnston, 1999). However, the subcellular localization and stability of *A. oryzae* CreA are not affected by disruption of the *SNF1* ortholog (Tanaka et al., 2018). This indicates that the regulatory mechanisms for the subcellular localization of yeast Mig1 and *A. oryzae* CreA are different. In agreement with this, the purified recombinant protein of a *T. reesei* Snf1 ortholog phosphorylates yeast Mig1 but not *T. reesei* Cre1, an ortholog of CreA (Cziferszky et al., 2003). In budding yeast, hexokinase Hxk2 is also phosphorylated by Snf1 and is involved in the regulation of Mig1 nuclear export (Ahuatzi et al., 2007). *Aspergillus* species have two functional glucose kinases, hexokinase HxkA and glucokinase GlkA (Fleck and Brock, 2010). It would be interesting to investigate the involvement of these glucose kinases in the nuclear export and degradation of CreA in *A. oryzae*.

Several recent studies have examined the phosphorylation of CreA in *A. nidulans*. Six serine residues (S262, S277, S288, S289, S312, and S319) in *A. nidulans* CreA were identified as phosphorylation sites by LC-MS analysis, and three of them (S289, S312, and S319) were phosphorylated only under conditions of growth in a glucose medium (Alam et al., 2017). Another recent study revealed six additional phosphorylation sites in *A. nidulans* CreA (S176, S268, S281, S284, T308, and S406; de Assis et al., 2021). The replacement of T308 with alanine significantly inhibited the nuclear accumulation of CreA-GFP. In contrast, the replacement of S262 or S268 with alanine increased the nuclear localization of CreA-GFP under de-repressing conditions, although these CreA-GFP mutants were not detectable by western blotting under such conditions (de Assis et al., 2021). Phosphorylation at S319 is lost upon the deletion of *pkaA*, which encodes a cAMP-dependent protein kinase, whereas CreA is not a direct target of this protein kinase (Ribeiro et al., 2019). Although the replacement of S319 with alanine has no significant effect on CreA subcellular localization, this mutation and the T308A mutation significantly increased CreA-GFP protein levels under repressing conditions (de Assis et al., 2021). However, none of the substitutions of these phosphorylation sites with alanine had a significant effect on CreA degradation under de-repressing conditions (de Assis et al., 2021). A recent study showed that replacement of S388 at the *T. reesei* Cre1 C-terminus with valine releases CCR (Han et al., 2020). This serine residue is conserved in the *Aspergillus* CreA proteins. However, deletion of 20 amino acids in the C-terminus, including this conserved serine residue, has no effect on the stability of *A. oryzae* CreA (Tanaka et al., 2018).

Identification of the protein kinase that phosphorylates CreA is an important issue that remains to be addressed. In *T. reesei*, S241 within Cre1 (corresponding to S262 in *A. nidulans* CreA) has been identified as a phosphorylation site, and the phosphorylation of this serine residue positively regulates DNA binding (Cziferszky et al., 2002). In addition, casein kinase II is a strong candidate kinase that phosphorylates Cre1 (Cziferszky et al., 2002). In *A. nidulans*, casein kinase CkiA has been identified as an interacting partner of Fbx23 (de Assis et al., 2018). Therefore, it is important to elucidate the role of casein kinase in the regulation of CreA function.

### Glucose-Induced Endocytosis of Maltose Transporter

The amount of membrane proteins, including transporters involved in nutrient uptake, is tightly controlled. When the extracellular environment changes, unnecessary membrane proteins are internalized into the cell by endocytosis and transported to the vacuole for degradation (Polo and Di Fiore, 2008). This endocytosis is caused by ubiquitination of cell membrane proteins. MalP is the major maltose transporter required for amylolytic enzyme production in *A. oryzae* (Hasegawa et al., 2010; Hiramoto et al., 2015). When glucose is added, MalP is translocated from the cell membrane to the vacuole *via* endocytosis (Hiramoto et al., 2015). The addition of mannose, which induces the CCR of amylolytic genes, as well as glucose, also induces MalP endocytosis (Hiramoto et al., 2015). These results suggest that MalP is degraded to inhibit the uptake of maltose, which induces the expression of amylolytic genes. CreD and HulA are essential for this glucose-induced endocytosis of MalP (Tanaka et al., 2017). Therefore, in the presence of glucose, CreD probably induces endocytosis by recruiting HulA to MalP. Although CreD is rapidly dephosphorylated upon the addition of glucose, the phosphorylation state of CreD is not associated with the interaction with HulA and the endocytosis of MalP (Tanaka et al., 2017). The regulatory mechanism by which CreD induces glucose-specific MalP ubiquitination is unknown. In budding yeast, ART proteins, including Rod1/Art4, are also ubiquitinated by Rsp5. This ubiquitination is required for adaptor activation or degradation (Ho et al., 2017; MacDonald et al., 2020). It is important to examine whether CreD is ubiquitinated in *A. oryzae*.

The inhibition of glucose-induced endocytosis is expected to enhance substrate uptake. In fact, cellobiose consumption and ethanol production were enhanced by stabilizing heterologously expressed cellobiose transporters in budding yeast lacking four ARTs, including Rod1/Art4 (Sen et al., 2016). However, disruption of *creD* in *A. oryzae* counteracts the release of CCR by *creB* disruption and reduces α-amylase production (Tanaka et al., 2017). Therefore, stabilizing MalP without *creD* disruption, e.g., by introducing mutations in the ubiquitination sites of MalP, is necessary to increase amylase production.

## Discussion

**Figure 1:**
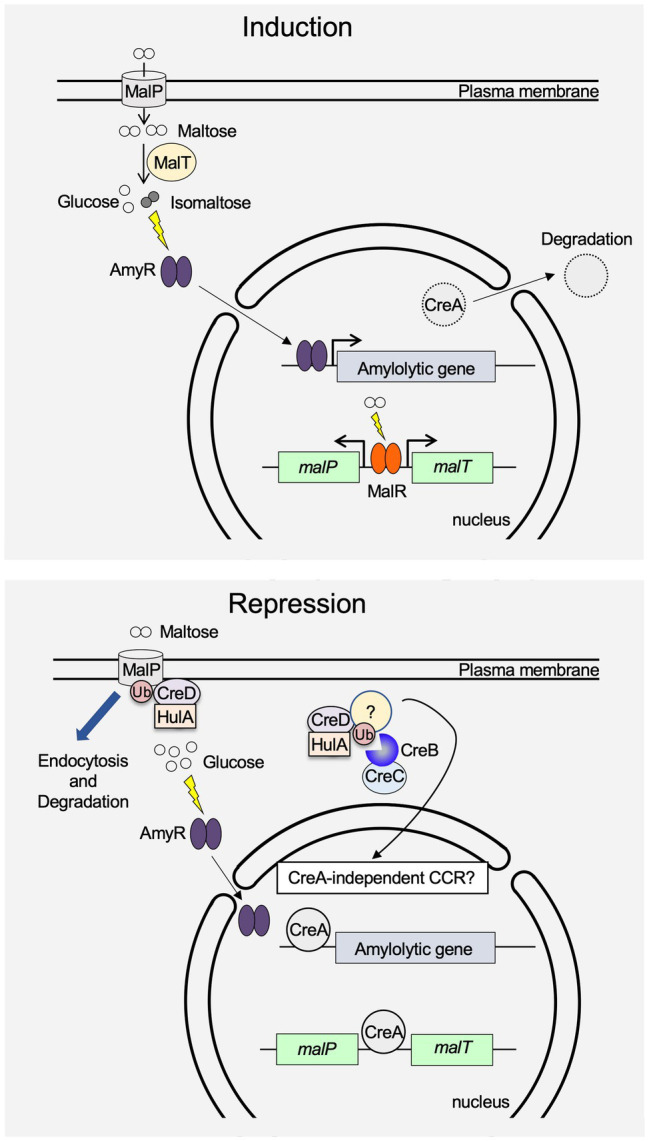
Schematic model for induction and repression of amylolytic gene expression in *A. oryzae*. When maltose is added to the culture medium, MalR induces *malP* and *malT* expression. After the uptake of maltose into the cell by MalP, AmyR nuclear transfer is triggered by isomaltose, which is presumably generated by the glycosyltransferase activity of MalT. CreA is exported from the nucleus to the cytoplasm, where it is degraded, and amylolytic gene expression is induced by activated AmyR. In the presence of glucose, the expression of *malP* and *malT* is repressed by CreA. Moreover, MalP protein is brought into the cell by endocytosis mediated by HulA and CreD, and is degraded at the vacuole. Although a high concentration of glucose induces AmyR nuclear transfer, expression of amylolytic genes is strongly repressed by CreA. The unknown protein stabilized by the CreB-CreC complex presumably regulates CreA-independent carbon catabolite repression (CCR).

As mentioned above, details on the regulatory mechanism of the induction and repression of hydrolytic gene expression in *A. oryzae* are becoming clearer, particularly for amylolytic genes (Figure 1). The release of CCR is significantly effective in improving the productivity of hydrolytic enzymes. In addition, constitutive activation of transcription factors leads to a reduction in the cost of producing hydrolytic enzymes (Alazi and Ram, 2018). However, the molecular mechanism of transcriptional regulation of hydrolytic enzyme genes in *A. oryzae* has not been elucidated, even for amylolytic genes. For example, *glaB* gene expression in solid-state culture requires at least AmyR and FlbC, but another unidentified transcription factor(s) seems to be needed because promoter deletion analysis shows that a GC-rich sequence is important for *glaB* expression (Ishida et al., 2000; Hisada et al., 2013). The AmyR-binding sequence and putative FlbC-binding sequence are different from this GC-rich sequence, to which a certain transcription factor may bind. Moreover, because recent studies have revealed that the regulatory mechanism of hydrolytic gene expression differs among *Aspergillus* species; further research on the regulatory mechanism of hydrolytic gene expression in *A. oryzae* should be accelerated.

Autolysis of the *A. oryzae* mycelium is important for its production of volatile compounds that contribute to soy sauce flavor (Xu et al., 2016). Cell wall degrading enzymes including chitinase and β-1,3-glucanase degrade fungal cell walls, and intracellular proteases and nucleases then degrade proteins and DNA/RNA, respectively, during autolysis. In *A. nidulans*, two transcription factors, RlmA and XprG, which regulate glucanase and chitinase genes, are involved in autolysis (Katz et al., 2013; Kovács et al., 2013). Given that no intracellular protease or nuclease gene expression regulatory mechanisms have been reported thus far, identification of transcription factors involved in *A. oryzae* autolyis will aid in development of industrial applications for *A. oryzae*. In addition, chitin in the *A. oryzae* cell wall adsorbs secreted α-amylase. This adsorption is inhibited when chitin is masked by α-1,3-glucan (Sato et al., 2011; Zhang et al., 2017). In this regard, regulatory mechanisms for genes encoding cell wall remodeling enzymes are also of interest.

The analysis of gene expression mechanisms in *A. oryzae* has progressed significantly after the completion of *A. oryzae* whole genome sequencing. In particular, similar to that for *A. fumigatus* (Furukawa et al., 2020), a disruption library for transcriptional regulatory genes, including transcription factors and transcription-related machinery, has been constructed by the Japanese *A. oryzae* research community. This disruption library as well as the overexpression library which covers a part of transcription factor genes has greatly contributed to the identification of novel transcription factors responsible for hydrolytic gene expression, such as FlbC, ManR, and PrtR. Such libraries have also been effectively utilized to mine novel transcription factors, such as AtrR (azole drug resistance and ABC transporter gene regulation; Hagiwara et al., 2017), EcdR (conidiophore development; Jin et al., 2011), and KpeA (kojic acid production and conidiation; Arakawa et al., 2019), that are involved in the regulatory expression of genes other than hydrolytic genes. Since the library of disruption mutant strains contains over 500 genes putatively involved in transcriptional regulation, further identification of novel transcription factors that regulate the expression of hydrolytic genes in *A. oryzae* is highly expected.

## Author Contributions

MT and KG wrote the manuscript. All authors contributed to the article and approved the submitted version.

### Conflict of Interest

The authors declare that the research was conducted in the absence of any commercial or financial relationships that could be construed as a potential conflict of interest.
